# 316. Use of (1-3)-β-D-Glucan Assay for Diagnosis of Candidemia in Patients Hospitalized with SARS-CoV-2 Infection

**DOI:** 10.1093/ofid/ofab466.518

**Published:** 2021-12-04

**Authors:** Yesha Malik, Amy Dupper, Jaclyn Cusumano, Dhruv Patel, Kathryn Twyman, Deena Altman, Dana Mazo, Rachel Chasan

**Affiliations:** 1 Icahn School of Medicine at Mount Sinai/Elmhurst, New York, New York; 2 Icahn School of Medicine at Mount Sinai, New York, NY; 3 Mount Sinai Queens Hospital, Queens, New York; 4 Icahn School of Medicine at Mount Sinai Hospital, New York, New York; 5 Mount Sinai Health System, New York, New York; 6 The Mount Sinai Hospital, New York, New York

## Abstract

**Background:**

Candidemia is a rare but serious complication of SARS-CoV-2 hospitalization. Combining non-culture and culture-based diagnostics allows earlier identification of candidemia. Given higher reported incidence during COVID-19 surges, we investigated the use of (1-3)-β-D-glucan (BDG) assay at our institution in those who did and did not develop candidemia.

**Methods:**

Retrospective study of adults admitted to The Mount Sinai Hospital between March 15-June 30 2020 for SARS-CoV-2 infection, with either ≥1 BDG assay or positive fungal blood culture. Data was collected with the electronic medical record and Vigilanz. A BDG value ≥ 80 was used as a positivity cutoff. Differences in mortality were assessed by univariate logistic regression using R (version 4.0.0). Statistical significance was measured by *P* value < .05.

**Results:**

There were 75 patients with ≥1 BDG assay resulted and 28 patients with candidemia, with an overlap of 9 between the cohorts. Among the 75 who had BDG assay, 23 resulted positive and 52 negative. Nine of 75 patients developed candidemia. Of the 23 with a positive assay, 5 developed candidemia and 18 did not. Seventeen of the 18 had blood cultures drawn within 7 days +/- of BDG assay. Four patients with candidemia had persistently negative BDG; 2 had cultures collected within 7 days +/- of BDG assay. With a cut-off of >80, the negative predictive value (NPV) was 0.92. When the cut-off increased to >200, NPV was 0.97 and positive predictive value (PPV) was 0.42. Average antifungal days in patients with negative BDG was 2.6 vs. 4.2 in those with a positive. Mortality was 74% in those with ≥1 positive BDG vs. 50% in those with persistently negative BDGs. There was a trend towards higher odds of death in those with positive BDG (OR = 2.83, 95% CI: 1.00-8.90, p < 0.06).

**Conclusion:**

There was substantial use of BDG to diagnose candidemia at the peak of the COVID-19 pandemic. Blood cultures were often drawn at time of suspected candidemia but not routinely. When cultures and BDG were drawn together, BDG had a high NPV but low PPV. High NPV of BDG likely contributed to discontinuation of empiric antifungals. The candidemic COVID-19 patients had high mortality, so further investigation of algorithms for the timely diagnosis of candidemia are needed to optimize use of antifungals while improving mortality rates.

**Disclosures:**

**All Authors**: No reported disclosures

